# Carbonic Anhydrase IX Inhibitors as Candidates for Combination Therapy of Solid Tumors

**DOI:** 10.3390/ijms222413405

**Published:** 2021-12-14

**Authors:** Stanislav Kalinin, Anna Malkova, Tatiana Sharonova, Vladimir Sharoyko, Alexander Bunev, Claudiu T. Supuran, Mikhail Krasavin

**Affiliations:** 1 Institute of Chemistry, Saint Petersburg State University, 199034 Saint Petersburg, Russia; st030565@student.spbu.ru (A.M.); st072335@student.spbu.ru (T.S.); v.sharoyko@spbu.ru (V.S.); m.krasavin@spbu.ru (M.K.); 2 School of Pharmacy, University of Eastern Finland, 70211 Kuopio, Finland; 3 Medicinal Chemistry Center, Togliatti State University, 445020 Togliatti, Russia; brglab@tltsu.ru; 4 Neurofarba Department, Universita degli Studi di Firenze, 50019 Florence, Italy; claudiu.supuran@unifi.it

**Keywords:** carbonic anhydrase IX, small-molecule inhibitors, solid tumors, tumor hypoxia, tumor acidosis combination cancer therapy, adjuvant agents

## Abstract

Combination therapy is becoming imperative for the treatment of many cancers, as it provides a higher chance of avoiding drug resistance and tumor recurrence. Among the resistance-conferring factors, the tumor microenvironment plays a major role, and therefore, represents a viable target for adjuvant therapeutic agents. Thus, hypoxia and extracellular acidosis are known to select for the most aggressive and resilient phenotypes and build poorly responsive regions of the tumor mass. Carbonic anhydrase (CA, EC 4.2.1.1) IX isoform is a surficial zinc metalloenzyme that is proven to play a central role in regulating intra and extracellular pH, as well as modulating invasion and metastasis processes. With its strong association and distribution in various tumor tissues and well-known druggability, this protein holds great promise as a target to pharmacologically interfere with the tumor microenvironment by using drug combination regimens. In the present review, we summarized recent publications revealing the potential of CA IX inhibitors to intensify cancer chemotherapy and overcome drug resistance in preclinical settings.

## 1. Introduction

Increasingly prevalent cancer treatment failure largely stems from anticancer drug resistance in tumors and represents one of the major challenges to the healthcare system in the XXI century. Despite the prominent development of novel approaches in the fields of surgery, radiation therapy, and laser therapy, it is a chemotherapeutical treatment that remains the main tool for the management of advanced-stage tumors [1]. Therefore, there is an urgent need for innovations overcoming the main causes of drug resistance, which include drug inactivation, drug target alteration, drug efflux, DNA damage repair, cell death inhibition, epithelial-mesenchymal transition, and inherent tumor cell heterogeneity [2]. While drug target alteration and tumor cell heterogeneity are still extremely challenging to combat, microenvironment-based tumor protective mechanisms appear more tractable in terms of pharmacological targeting [3]. Particularly, poor oxygen supply and hypoxia-related acidosis are widespread phenotypic characteristics of many malignancies. Furthermore, these two features are often associated with poor prognosis as they select for highly aggressive, stem cell-like phenotypes that are non-responsive to the hitherto efficient treatments [4]. This results in a rapid tumor recurrence and threatens patients’ survival [5]. In light of these facts, combining anticancer drugs with adjuvant agents targeting tumor protective mechanisms is turning into a widely acknowledged approach and treatment standard. It is noteworthy that hypoxia-related acidosis attracts immediate attention in this context, contributing to drug inactivation, as well as metabolic adaptations in highly malignant cell phenotypes. Moreover, beyond tumor survival, hypoxia and acidosis largely enable cancer cell invasion, migration, and metastasis, which drastically complicates tumor management, and thus, therapeutic interventions disrupting these mechanisms are of great interest [6,7]. Noteworthy, due to its cancer-specific tissue localization, carbonic anhydrase IX (CA IX), a pH-regulating enzyme overexpressed in hypoxic niches, holds great promise as a targetable protein associated with highly malignant and stem cell-like cancer phenotypes [8].

Carbonic anhydrases (CA) are ubiquitous zinc metalloenzymes that catalyze CO_2_–HCO_3_^−^ interconversion, therefore, governing a major buffering system across cell compartments and in extracellular space. While twelve catalytically active isoforms are present in human tissues, it is the membrane-anchored CA IX isoform that displayed a clear association with tumor cells, being overexpressed in hypoxia regions where it has a major impact on intracellular pH (pH_i_)/extracellular pH (pH_e_) regulation [8,9,10].

Interestingly, since the active sites are highly conservative among the isozymes, developing type-specific CA inhibitors (CAIs) remains a challenging task. In fact, sulfonamide-based drugs, which are used in the clinic as diuretics and glaucoma treatments (where cytosolic CA II is a main pharmacological target), are generally pan-isotype CA inhibitors. On the other hand, highly potent and CA-IX-selective small molecules emerged in the last decades, with SLC-0111 recently progressing to Phase Ib/II clinical study, as well as its sulfamate analogs with favorable inhibitory profiles (for CA inhibitors discussed in the present see Table 1) [11,12].

It should also be noticed that CA IX-targeting immunotherapeutics have been reported and contributed significantly to deciphering CA IX’s role in tumor pathogenesis. Furthermore, CA IX-targeting antibodies have entered clinical trials, albeit their efficacy remains unclear largely due to poor perfusion of hypoxic tumor areas where CA IX is overexpressed [18]. Noticeably, CA IX-targeting molecules recommended themselves for radiotherapy and transonic applications [19,20,21,22,23].

CA IX overexpression in cancer cells is associated with hypoxia and poor vascularization of tumor mass [24]. In such conditions, glycolytic metabolic reprogramming occurs, implying an accumulation of acidic species, such as lactate, and a significant decrease of pH_i_ [25]. Hypoxia and accompanying intracellular space acidification activate the HIF-1α (hypoxia induced factor 1α) pathway leading to the upregulation of hypoxia response element (HRE) and CA IX expression [26]. In turn, CA IX interferes with membrane transport proteins, thus alleviating the intracellular acidosis and allowing for acidification of extracellular medium [27]. Therefore, emerging tumor acidosis is a crucial feature of the tumor microenvironment, which contributes significantly to the poor therapeutic outcomes and drug resistance [28]. Particularly, the following effects have been described:

- decreasing drug uptake in tumor cells due to the altered protonation state;
- selecting for highly aggressive and drug-resistant phenotypes possessing stem cell characteristics, and harboring protumorigenic mutations associated with poor treatment response;
- enhancing invasion and metastatic processes through apoptosis induction in surrounding cells, elevated secretion of proteinases, and the disruption of E-cadherin-mediated cell adhesion;
- inducing angiogenesis via activating VEGF (vascular endothelial growth factor) production;
- decreasing immune infiltration of tumors.

In this context, multiple roles of CA IX in tumor survival and development have been identified in recent decades, and these aspects have been reviewed elsewhere [8,29].

An increasing number of reports currently implicates the role of CA IX blocking in cancer cells in intensifying standard chemotherapy of solid tumors, as well as overcoming ubiquitously encountered acidosis-promoted drug resistance. Furthermore, impeding hypoxia-driven glycolytic metabolism through blocking pHi alkalization in highly aggressive phenotypes is considered a powerful tool for preventing tumor cell invasion and metastasis [30]. Despite the great promise held by the drug combinations involving CA IX inhibitors, the number of in-depth studies on such treatments remains very limited, as highly justified models of the tumor microenvironment are required to perform conclusive investigations.

Herein we summarized recent research publications implicating malignant cells’ exposure to combinations of CAIs with either conventional cytostatic agents or targeted anticancer drugs. Concise mechanistic context and essential experimental details are provided where possible. Thus, the present review serves to increase researchers’ interest in CA IX inhibitors as anticancer adjuvant agents and highlights the lessons learned from the recently reported preclinical studies.

## 2. Carbonic Anhydrase Inhibitors/Anticancer Agent Combinations

### 2.1. Acetazolamide in Combination with Conventional Cytostatic Agents

#### 2.1.1. Acetazolamide and Doxorubicin

**Figure 1:**
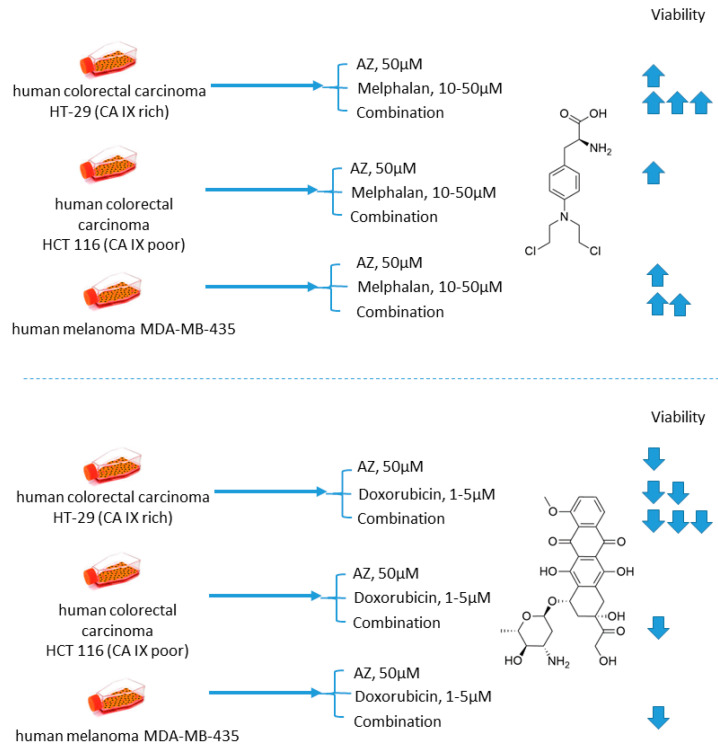
Graphical outline of the study on AZ+melphalan/DOX combination reported by Geiling et al. [33].

Cellular uptake of a wide range of conventional cytostatic agents can be hindered due to the shifts in tumor microenvironment characteristics, such as poor perfusion, hypoxia, and/or acidity [31]. The widely acknowledged ion-trapping model predicts that the decrease in extracellular pH, which is often encountered in hypoxic regions, prevents weekly basic drugs from penetrating malignant cells and thus confers regional failure [31]. Inhibition of CA IX isoform on the cellular surface proved to be a viable approach for affecting extracellular acidification in solid tumors [32]. One of the pioneering attempts to employ small molecular CA IX blockers to overcome cytostatic resistance in hypoxic (acidic) regions was made in 2012 by Geiling and colleagues who investigated the combined effect of CA pan-inhibitor acetazolamide (AZ) and doxorubicin (DOX) [33]. To this end, the authors used colon carcinoma HT-29 (CA IX rich) and HCT 116 (CA IX poor) cell lines, as well as MDA-MB-435 human melanoma cells, stably transfected to express empty vector (EV1) or CA IX (CA9/18) (Figure 1). Interestingly, the treatment with AZ significantly increased the toxicity of DOX in CA IX-rich cells. Conversely, DOX efficacy was unaffected by AZ treatment in the cells with low CA IX expression. The authors concluded that the effect of AZ treatment is largely related to the blocking of CA IX, which is instrumental for cancer cells to maintain acidic pH_e_, thus hampering the membrane transport of weakly basic drugs, including DOX. This point was further supported by the fact that the weakly acidic drug melphalan exerted reduced toxicity under the same conditions against the CA IX-rich cell lines (Figure 1) [33].

#### 2.1.2. Acetazolamide and Sulforaphane

Mokhtari and coworkers reported a series of studies investigating the synergistic activity of AZ and natural product sulforaphane against bronchial carcinoid cell lines and their derived mice subcutaneous xenografts [34]. Sulforaphane shows multiple anticancer effects [35,36,37]. Among others, it selectively activates the nuclear transcription factor erythroid 2p45 (NF-E2)-related factor 2 (Nrf2)-Kelch-like ECH-associated protein 1 (Keap1), which is an essential downstream effector of the PI3K/Akt/mTOR [38,39]. Importantly, overexpression of Nrf2 due to defects in Keap1 was observed in several types of cancer, where it served as a non-HIF mediated mechanism, promoting tumor cell survival in hypoxic conditions, including apoptosis inhibition, metabolic reprogramming, and chemotherapeutic resistance [40]. Finally, sulforaphane has been reported to reduce the expression of serotonin receptors (5-HT2, 5-HT3) and transporter (SERT) in Caco-2 cells, which appears relevant with regard to the treatment of hormone-secreting bronchial carcinoids [41,42].

As unveiled by Mokhtari, combined treatment of NCI-H727 (typical) and NCI-H720 (atypical) lung carcinoid cells with AZ and sulforaphane (SFN) led to a significant reduction in their viability and clonogenicity, as well as markedly decreased the fraction of invasive cells compared to the single-agent controls. In addition, a profound drop in the tumor-derived serotonin has been noticed in vitro. The authors suggested that the observed synergistic effects could be explained through the inhibition of both MEK/Erk (MAPK/ERK kinase/ extracellular signal-regulated kinase) and PI3K/Akt (phosphatidylinositol-3 kinase/Akt) pathways, which in turn regulate the expression of CA IX and other HIF-targeted genes [34]. Furthermore, in H727 and H720 spheroids, the AZ+SFN combination largely decreased proliferation rates compared to single-agent therapies. Noticeably, when xenografted into immunocompromised mice, these spheroids still strongly responded to the combined treatment, and a prominent reduction in tumor growth and gross vascularization was observed. Such effect was accompanied by a remarkably decreased expression of the stemness markers. The in vivo studies also further confirmed the downregulation of the PI3K/Akt/mTOR (mechanistic target of rapamycin) pathway and downstream effectors, as well as a more profound perturbation of pH homeostasis in lung cancers under exposure to AZ+SFN treatment. Moreover, these conditions markedly reduced the 5-HT expression in both the atypical H720 and typical H727 bronchial carcinoid xenograft, and the highly aggressive atypical histotype was extremely sensitive to the treatment. Therefore, the combination AZ+SFN promisingly exerted multiple anticancer agents in different models of bronchial carcinoid via blocking a range of hypoxia-mediated pro-survival pathways and 5-HT secretion in bronchial tumors [43].

**Figure 2:**
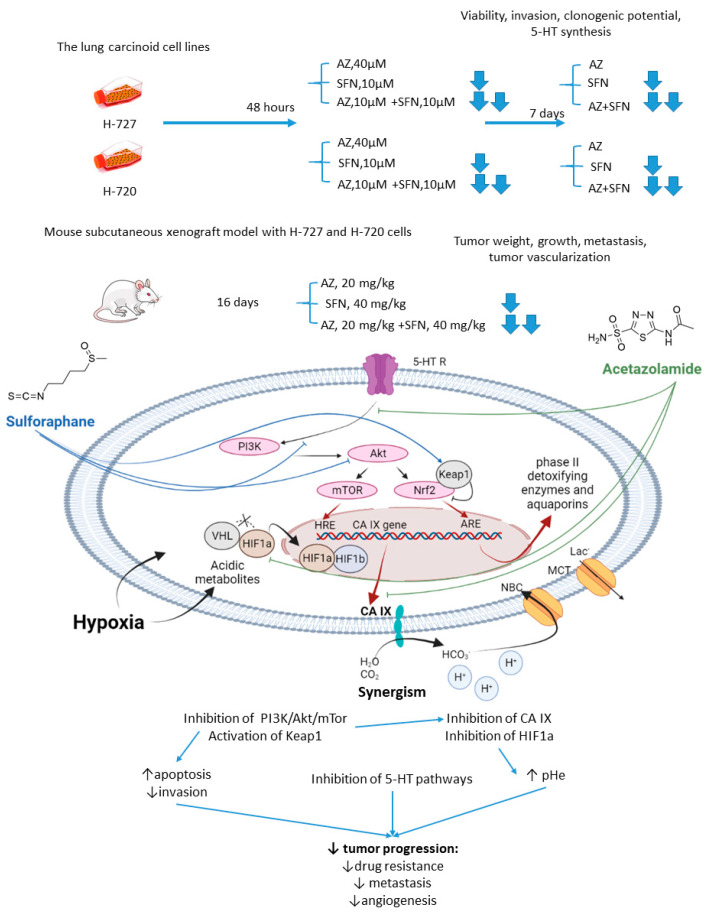
Graphical outline of the study on AZ+SFN combination reported by Mokhtari et al., and summary on the hypothesized molecular interactions resulting in synergistic action of the drugs [43,44].

In 2017, similar experiments with SFN+AZ combination were performed against HTB-9 and RT112(H) human bladder tumor cell lines and in vivo on the corresponding xenografts [44]. SFN+AZ treatment produced a substantial decrease in the cancer cell viability, growth, invasion, clonogenic potential, and apoptosis. In line with these in vitro results, the drug combination reduced the tumor growth in xenograft-bearing mice. Subsequent biochemical experiments revealed that co-administration of SFN and AZ caused downregulation of the PI3K/mTOR pathway in bladder tumors. Moreover, the metastatic burden was significantly decreased in animals, which was associated with decreased expression of E-cadherin, N-cadherin, and vimentin. Summarized, the joint action of the drugs against highly aggressive bronchial and bladder cancers was associated with restored pH_i_ and pH_e_ values and a more efficient blockade of PI3K/Akt/mTOR pathway as compared to single-agent treatment. In addition, the combined treatment enhanced apoptosis and produced a significant drop in the expression of adhesive molecules and stemness markers in tumor xenografts, whereas the expression of 5-HT was profoundly affected in the pulmonary carcinoid (Figure 2).

#### 2.1.3. Acetazolamide and Cisplatin

**Figure 3:**
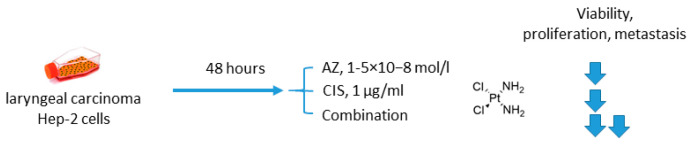
Graphical outline of the study on AZ+CIS combination reported by [46].

Another study involving AZ was reported by Gao in 2018 [45]. In this case, AZ was combined with an old DNA-alkylating drug cisplatin (CIS) to treat Hep-2 laryngeal carcinoma cells (Figure 3). The authors showed that the suggested drug combination was more efficient than single-agent treatment and resulted in decreased levels of Hep-2 cell viability, proliferation, and metastasis. Meanwhile, elevated expression of p53 and drop in the Bcl-2/bax ratio corresponded to higher rates of apoptosis in cancer cells. Unexpectedly, in this study, healthy human umbilical vein endothelial cells (HUVEC) turned out to be insensitive to both AZ and CIS either in single-agent or combined regimens, yet there are no evident reasons for such selectivity (at least in the case of cisplatin) (Figure 3) [45].

### 2.2. Acetazolamide in Combination with Targeted Anticancer Drugs

#### 2.2.1. Acetazolamide and Rapamycin

**Figure 4:**
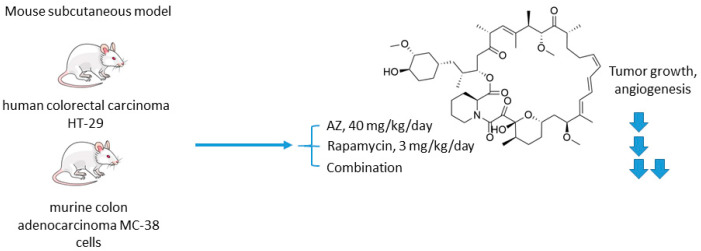
Graphical outline of the study on AZ+rapamycin combination reported by Faes et al. [50].

The mechanistic target of rapamycin (mTOR) is a serine/threonine-specific protein kinase that belongs to the family of phosphatidylinositol-3 kinase (PI3K) [46]. Participating in multiple signaling pathways by forming mTOR complexes 1 and 2 (mTORC1 and mTORC2), this protein regulates cell cycle progression, apoptosis, autophagy, proliferation, and metabolism of tumor cells [47]. Macrolide’s compound rapamycin and its analogs (also known as rapalogs) represent the first generation mTOR inhibitors [48]. Despite showing encouraging results in preclinical models as monotherapeutic agents, these drugs demonstrated limited efficacy in patients as tumor relapse often occurred through resistance formation [49]. In 2016, Faes and coworkers reported that the activity of mTORC1 is mainly restricted to the non-hypoxic tumor compartment, suggesting that there is a potential for combination treatment involving hypoxia-targeting molecules (Figure 4) [50]. In fact, in vivo experiments on mice injected with colorectal carcinoma HT-29 cells and murine colon adenocarcinoma MC-38 cells highlighted that mTORC1 hyperactivation promotes tumor-cell proliferation in normoxia. Contrastingly, in hypoxic areas of the tumor, a decrease in mTORC1 activity was observed, which abrogated rapamycin antitumor efficacy in these areas. Furthermore, rapamycin treatment increased the hypoxic tumor compartment compared to controls in both HT-29 tumor xenografts and MC-38 tumor allografts and gave significant rise to CA IX protein expression in these regions. Based on these findings, the authors subsequently evaluated the joint effect of AZ and rapamycin in the mouse models [50]. It turned out that both AZ and rapamycin alone reduced tumor growth; however, the effect was dramatically increased when both agents were combined. Interestingly, the observed effect was long-lasting, as after three months of the treatment HT-29 tumor xenografts did not progress. It was additionally demonstrated that AZ increased tumor necrosis and the number of tumor blood vessels in HT-29 and MC-38 tumors. Moreover, AZ drastically reduced proliferation in hypoxic, but not in normoxic tumor regions, whereas the opposite was true for rapamycin. Thus, combined administration of rapamycin and AZ produced remarkable antiproliferative effects in vivo in both hypoxic and non-hypoxic compartments (Figure 4) [50].

#### 2.2.2. Acetazolamide and Bevacizumab

**Figure 5:**
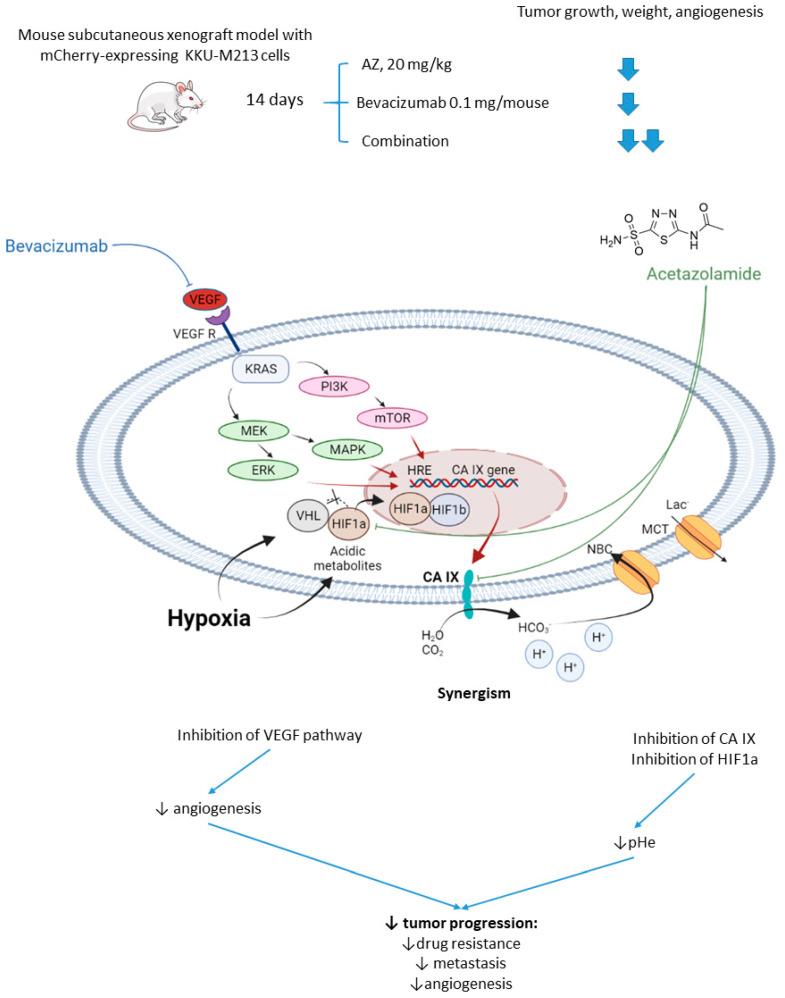
Graphical outline of the study on AZ+bevacizumab combination reported by Vaeteewoottacharn et al. and a summary on the hypothesized molecular interactions resulting in synergistic action of the drugs [51].

Vaeteewoottacharn and colleagues investigated AZ as an adjuvant agent for the treatment of cholangiocarcinoma, which has a very poor prognosis and a small range of therapy opportunities [51]. The overexpression of VEGF in tumor tissue gives rise to the use of VEGF inhibitors for this cancer; however, cancer cells’ adaptation to the treatment resulted in limited efficacy in patients [52]. Of note, HIF-1α and CA IX upregulation has been reported to contribute to the drug resistance against anti-angiogenic therapy [53]. In this context, Vaeteewoottacharn combined AZ with bevacizumab, a monoclonal antibody that blocks angiogenesis by inhibiting VEGF to treat cholangiocarcinoma tumors in vivo. Despite the fact that bevacizumab (0.1 mg/kg/mouse) effectively inhibited tumor growth, remarkable overexpression of HIF1α, VEGF, VEGFR1, and CA IX was observed in the treated tumors (Figure 5). The results, therefore, highlighted the compensative mechanism of the tumor in response to the VEGF inhibition. Reassuringly, the combination treatment with AZ produced a more significant reduction in tumor growth and angiogenesis, although the influence of the combined treatment on VEGF and HIF1α pathways remained uninvestigated (Figure 5) [51].

#### 2.2.3. Acetazolamide and Imatinib

**Figure 6:**
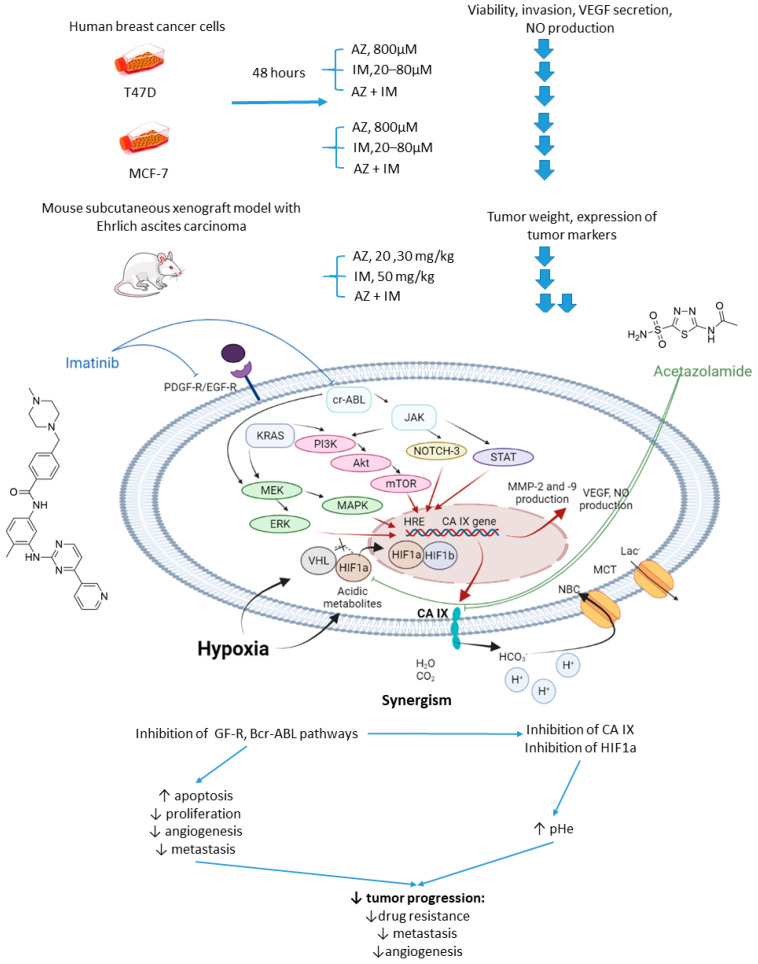
Graphical outline of the study on AZ+IM combination reported by Abd-El Fattah et al., and summary on the hypothesized molecular interactions resulting in the synergistic action of the drugs [54].

In 2017, Abd-El Fattah and colleagues combined imatinib (IM) with AZ in attempts to enhance the cellular uptake of the former weakly basic drug (Figure 6) [54]. IM is a tyrosine kinase inhibitor capable of blocking tumorigenic and prometastatic kinases Bcr-ABL, c-Kit, platelet-derived growth factor receptor (PDGFR), and epidermal growth factor receptor (EGFR) [55]. Co-administration of AZ led to a significant increase in the uptake of IM by cells T47D and MCF-7 breast cancer cells. The authors also reported considerable biochemical alterations in the presence of AZ compared to IM monotherapy. In particular, they observed suppression of HIF-1α mRNA, accompanied by a decrease in VEGF secretion, inhibition of NO release, a profound suppression of matrix metalloproteinases MMP-2 and 9 and phospho-p38 MAPK (mitogen activated protein kinase) and rise in tissue inhibitor of metalloprotease-1, 2 (TIMP-1, 2) levels. These results highlighted the potential of the drug combination in question to block angiogenesis and metastatic potential in solid tumors by affecting multiple signaling pathways. Subsequently, in vivo experiments were performed by using Ehrlich ascites carcinoma (EAC)-bearing mice. It was found that single-agent IM administration caused reduced tumor volume by almost 46% after 3 days of treatment. In the combination regimen, the tumor volume reduction amounted to 63%. Histopathological studies showed that MVD, Ki-67, VEGF, and CD34 expression levels were significantly increased in the isolated tumor specimens of EAC. No impact of IM on the CA IX expression in vivo and in vitro was detected in this work. These facts led to the conclusion that co-administration of AZ can significantly extend the anti-angiogenic and anti-metastatic effects of IM therapy (Figure 6) [54].

#### 2.2.4. Acetazolamide and MS-275

**Figure 7:**
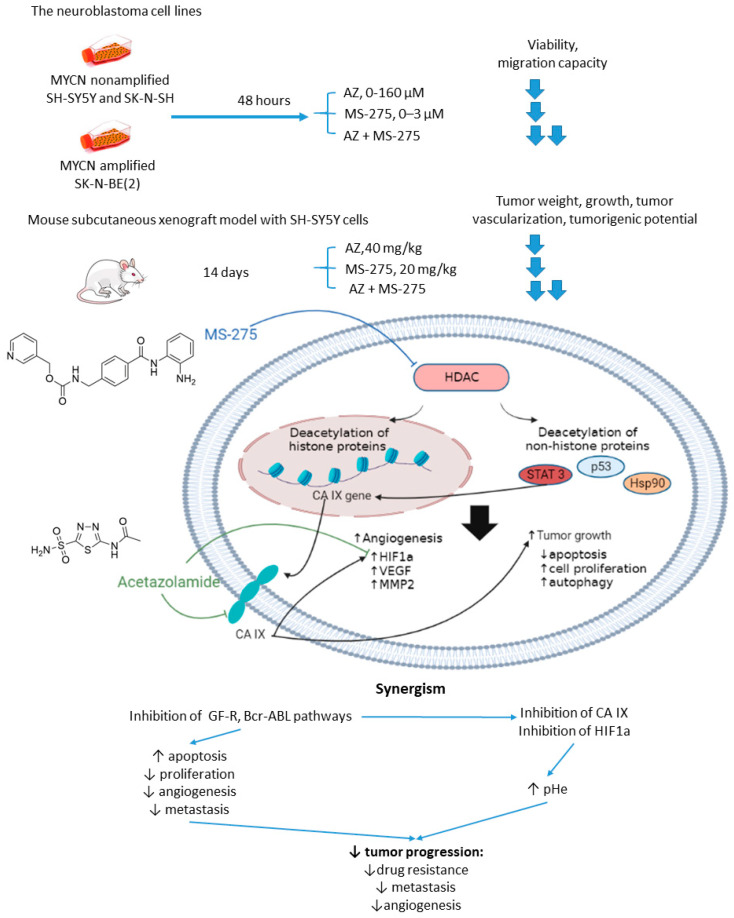
Graphical outline of the study on AZ+MS-275 combination reported by Mokharti et al., and summary on the hypothesized molecular interactions resulting in synergistic action of the drugs [60].

Overexpression or aberrant recruitment of histone deacetylases (HDACs) to the promoter of tumor-suppressor genes is one of the most common epigenetic alterations in tumor onset and progression [56]. Small molecule HDAC inhibitors, despite eliciting impressive results in preclinical settings, show limited efficacy in patients due to their toxic effects and emergence of drug resistance in cancer cells [57]. Therefore, continuous efforts are being made to discover drug combinations that would allow for achieving the full therapeutic potential of HDAC inhibitors in patients [58]. Of note, both CA IX and HDACs are overexpressed in neuroblastoma [59]. In the light of these facts, Mokhtarti and coworkers provided a study comparing the efficacy of MS-275, a small-molecule HDAC inhibitor, AZ, and their combination within in vitro and in vivo models of neuroblastoma (Figure 7) [60]. Intriguingly, co-administration of AZ and MS-275 led to a stronger decrease in cell viability and migration capacity as compared to the monotherapies. In turn, exposure of neuroblastoma SH-SY5Y xenografts to the combined AZ+MS-275 treatment yielded a significant reduction in tumor growth and vascularization. The authors reported a profound decrease in the expression of stemness markers in the tumors subjected to the AZ+MS-275 combination. Extensive apoptosis has been shown in the treated tumors, and the drug combination helped recover the T-cell balance. Finally, the combined treatment also led to a substantial downregulation of HIF1-α and CA IX, thereby confirming the contribution of AZ to the observed effects (Figure 7) [60].

### 2.3. Acetazolamide in Multidrug Combinations

#### Acetazolamide and CHOP Combination

CHOP is a chemotherapy combination that is used to treat non-Hodgkin lymphoma [61]. It includes cyclophosphamide, doxorubicin hydrochloride (hydroxydaunorubicin), vincristine sulfate (Oncovin), and prednisone [62]. Lymphomas are often marked with significant intratumor metabolic heterogenicity, which largely improves their therapy resistance [63]. In particular, hypoxic and highly acidified compartments tend to show a poor response to CHOP medication [64].

**Figure 8:**
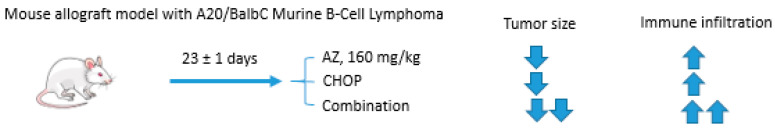
Graphical outline of the study on AZ+CHOP combination reported by Mehes et al. [65].

In 2020, Mehes and colleagues published an article unveiling benefits from co-administration of AZ as an adjuvant agent with CHOP medication in lymphomas (Figure 8) [65]. A simultaneous study of tumor progression together with tumor–host immune mechanisms was provided by using a recently established murine aggressive lymphoma model applying cultivated A20 B-cells [66]. Upon the six days of treatment with a single CHOP/single AZ intravenous injection, the size of hypoxic areas within the tumor decreased prominently. An even more massive drop in the size of the hypoxic compartments was observed when a single CHOP + five-day extended AZ administration regimen was used within the six days period. Both treatments caused a dramatic decrease in tumor size. Subsequent western blot analysis of the dissected tumor tissues revealed no increase in the CA IX levels upon treatment. Interestingly, as the immune system of the host animals remained intact in the performed model, the authors were able to demonstrate remarkable differences of CD3+ and CD8+ T-cell tumor infiltration in the analyzed specimens. In fact, minimal infiltrates were demonstrated in the control lymphoma samples, whereas combinations of CHOP and AZ raised the amount of the immune infiltration by one order of magnitude. Thus, CD3+ and CD8+ T-cell immune infiltration proved to be significantly higher under any of the AZ+CHOP adjuvant therapies compared to that of the control, CHOP-alone, as well as the AZ-alone, treated groups. Since it is widely acknowledged that tumor microenvironment acidification gains special importance for both drug availability and tumor–host immune interactions, the authors concluded that it was CA IX inhibitory activity of AZ that intensified the tumor-reductive effect of the conventional CHOP treatment and allowed for a prominent immune T-cell infiltration of tumors in vivo. Additionally, the local vasodilation effect exerted by AZ could be beneficial for the delivery of cytotoxic substances and immune effectors in animals [67]. Therefore, CHOP chemotherapy potentiation by AZ and the interplay between CA-inhibition and antitumor immune infiltrates in lymphomas were reported for the first time in this intriguing manuscript (Figure 8) [65].

### 2.4. Methazolamide in Combination with Conventional Cytostatic Agents

#### Methazolamide and Gemcitabine

**Figure 9:**
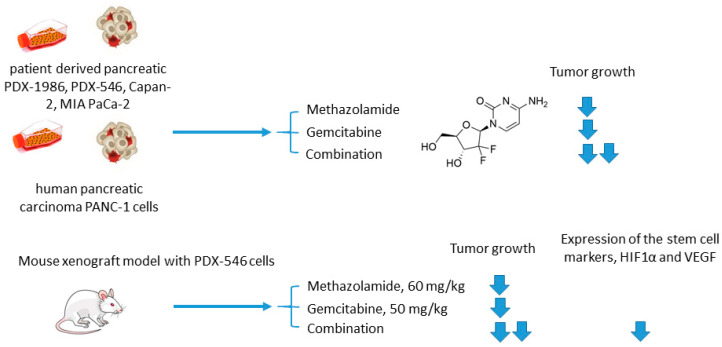
Graphical outline of the study on methazolamide+gemcitabine combination reported by Joshi et al. [68].

Joshi and colleagues employed another clinically used pan-isoform CAI methazolamide with a hope to intensify gemcitabine treatment of pancreatic cancers (Figure 9) [68]. In the reported in vitro experiments, Joshi and coworkers tested methazolamide+gemcitabine drug combination against patient-derived pancreatic carcinoma cells PDX-1986, PDX-546, Capan-2, MIA PaCa-2, as well as immortalized PANC-1 cells cultured in 2D and 3D modes [68]. Intriguingly, significant growth inhibition was produced by the drug combination in all cases compared to drug-alone controls. This encouraged the authors to further test the combined medication regimen in PDX-546-bearing mice. Reassuringly, the combination group showed more significant tumor growth inhibition compared to the drug alone. Meanwhile, the authors observed no detectable bodyweight loss suggesting good tolerance of the treatment. Histopathological studies revealed a pronounced reduction in expression of the stem cell markers and proliferation marker Ki-67 in the combination group, but not in drug-alone treated animals. In addition, both methazolamide and combination groups displayed anti-angiogenic morphological changes (would healing, tube formation), as well as a significant decrease in HIF1α and VEGF expression levels, highlighting the role of CAIs in the suppression of vascular growth in the tumor. Therefore, the potential of the methazolamide+gemcitabine combination for the treatment of pancreatic cancer has been preliminarily demonstrated (Figure 9) [68].

### 2.5. Isoform Selective Carbonic Anhydrase IX Inhibitors in Combination with Conventional Cytostatic Agents

#### 2.5.1. SLC-0111 and Dacarbazine/Temozolomide/Doxorubicin/5-Fluorouracil

Andreucci and coworkers performed a detailed in vitro study on the potentiation of cytotoxic agents’ efficacy in the presence of SLC-0111, a CA IX isoform-selective inhibitor that recently entered Phase Ib/II clinical trials [69]. To this end, human melanoma A375-M6, breast carcinoma MCF7, and colorectal carcinoma HCT 116 cell lines were employed.

**Figure 10:**
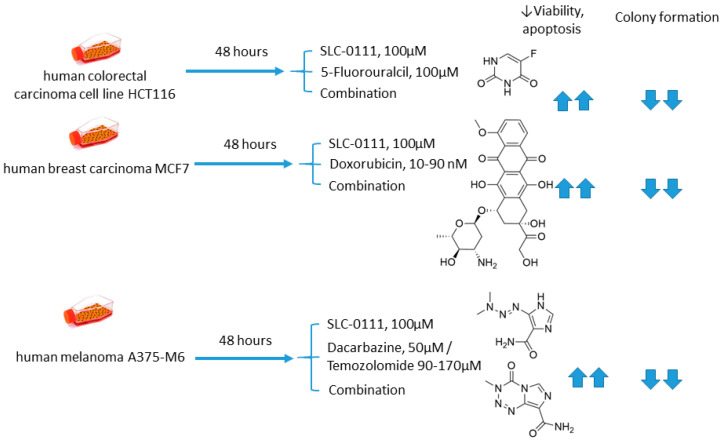
Graphical outline of the study on SLC-0111+dacarbazine/temozolomide/DOX/5-fluorouracil combination reported by Andreucci et al. [69].

The authors demonstrated that SLC-0111 markedly augmented cell death percentage, late apoptosis, and necrosis in A375-M6 melanoma cells when co-administered with guanine methylating agents (dacarbazin or temozolomide). A similar effect was produced by the SLC-0111+DOX combination against MCF7 breast cancer cells. In addition, all combinations efficiently blocked cell colony formation. This was not true, however, for the combination of SLC-0111 and 5-fluorouracil, which did not affect the cell viability of HCT116 colorectal carcinoma cells. Thus, SLC-0111 displayed the potential to sensitize cancer cells to conventional cytostatic agents, which was especially true for weakly basic drugs, but not for the weak acid 5-fluorouracil (Figure 10) [69].

#### 2.5.2. SLC-0111 and Temozolomide

**Figure 11:**
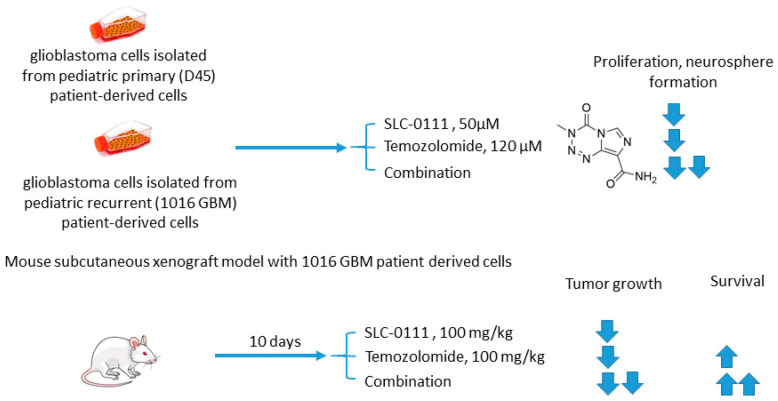
Graphical outline of the study on SLC-0111+temozolomide combination reported by Boyd et al. [70].

Boyd and colleagues investigated SLC-0111 for its ability to enhance the efficacy of temozolomide against glioblastoma in vitro and in vivo (Figure 11) [70]. The study revealed that monotherapy with SLC-0111 or temozolomide significantly decreased the growth of glioblastoma cells isolated from pediatric primary (D456) and a recurrent (1016 GBM) patient-derived xenograft in normoxia and hypoxia, whereas the combination caused a further drop in the cell growth but did not increase the toxicity of temozolomide against healthy astrocytes. It was shown that the SLC-0111+temozolomide combination prominently induced cell cycle arrest via DNA damage and lowered intracellular pH in cancer cells. Furthermore, the drug combination in question was highly efficient in vivo when administered to nude mice implanted with a 1016 GBM patient-derived xenograft. In fact, SLC-0111+temozolomide produced noticeable tumor regression in xenografts, and this effect was clearly greater than that of the drugs alone. Importantly, 130 days after xenograft implantation, the median survival for the temozolomide-alone treatment group was 76 days, while median survival could not be determined for the combination group. Analysis of tumor sections from the treated mice demonstrated that expression of the Brain Tumor Initiating Cell (BTIC) marker Sox2 was decreased with the co-administration of SLC-0111 with temozolomide, suggesting that SLC-0111 was capable of decreasing BTIC enrichment after temozolomide therapy. Therefore, the results obtained by Boyd highlight a significant benefit of the addition of SLC-0111 to the conventional temozolomide-based treatment for glioblastoma (Figure 11) [70].

#### 2.5.3. SLC-0111 and Gemcitabine

**Figure 12:**
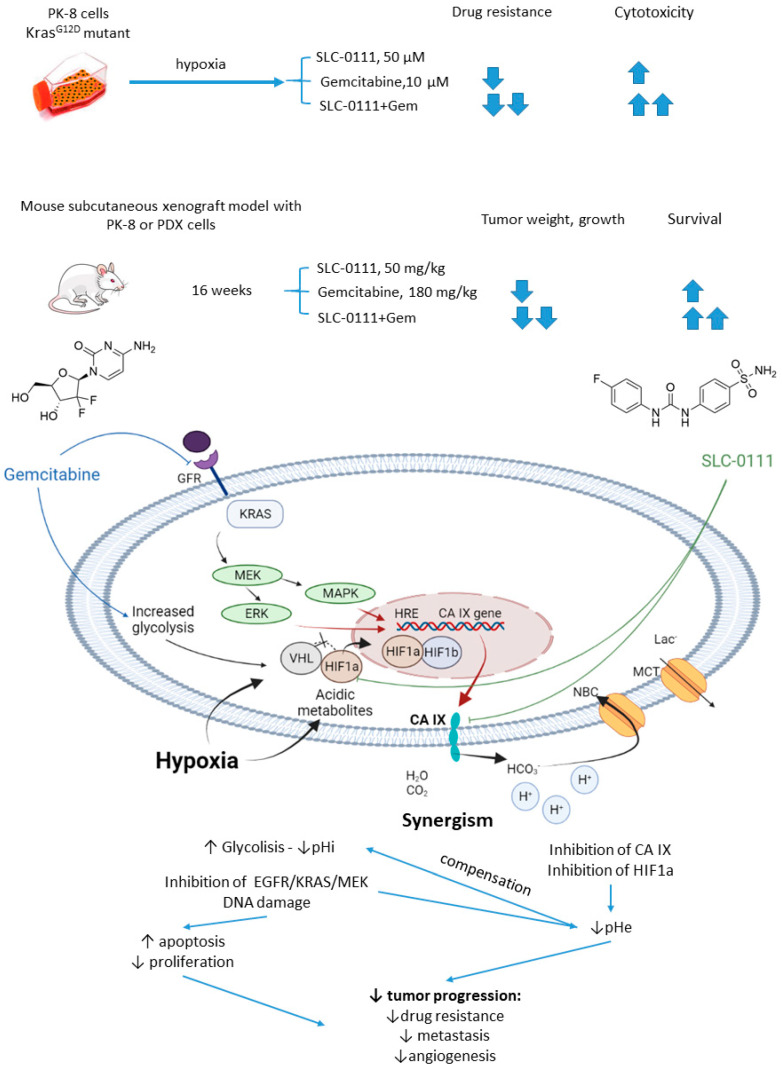
Graphical outline of the study on SLC-0111+gemcitabine combination reported by McDonald et al., and summary on the hypothesized molecular interactions resulting in synergistic action of the drugs [71].

In 2019, McDonald and colleagues evaluated the potential of SLC-0111 to improve therapeutic outcomes in pancreatic ductal adenocarcinomas expressing an activated form of Ki-ras2 Kirsten rat sarcoma (KRAS) oncogene (Figure 12) [71]. Approximately 93% of pancreatic adenocarcinomas harbor mutant KRAS oncogene, which drives tumor pathogenesis [72]. Intriguingly, extensive in vitro and in vivo studies performed by the authors revealed that KRAS-driven pancreatic ductal adenocarcinomas display a dependency on glycolysis and the need to buffer intracellular pH through the bicarbonate producing activity of CA IX. Thus, CA IX was identified as a pharmacologically targetable vulnerability downstream of mutant KRAS, acting as a hypoxia/pH-specific effector induced by the oncogene. In light of these facts, it became of utmost interest to evaluate the potential of CA IX inhibitors to sensitize KRAS mutant cells to chemotherapeutic agents, specifically to gemcitabine, which is typically used against this type of cancer. McDonald and coworkers reported significant intracellular pH drop and decreased survival rates in pancreatic cancer cells exposed to SLC-0111+gemcitabine treatment. In CA IX-positive KRAS-mutant PaCa83–2 patient-derived xenografts, 16 weeks of administration of the drug combination resulted in a dramatic increase in survival, as 100% of mice given the combination were alive, with one animal remaining tumor-free after the treatment. Finally, a histopathological study of Kras^G12D^/Pdx1-Cre/p53/Rosa^YFP^ genetically engineered mouse model revealed that after 14 days of treatment, the combination group displayed significantly fewer B220+ B-cells compared to control and single agents, which can be considered beneficial in the context of recent reports on B-cells promoting pancreatic tumorigenesis [73]. Meanwhile, no significant impact on the number of CD3+ T-cells was observed. Therefore, while suppressing tumor growth, glycolytic metabolic adaptation, and increasing survival rates in vivo, the drug combination did not have an adverse impact on the immune microenvironment (Figure 12) [71].

#### 2.5.4. S4 and Doxorubicin

Inspired by the acetazolamide-induced intensification of DOX treatment in CA IX-rich cell lines reported by Gieling et al. (*vide supra*), Kuijk and colleagues performed a follow-on study by using an isoform-selective CA IX inhibitor S4 [74]. This ureido-substituted sulfamate SLC-0111 analog has been earlier described to exert significant antiproliferative efficacy in vitro in different breast cancer tumor models [75,76]. It should be noticed, however, that despite encouraging in vitro results, the in vivo efficacy of S4 remained ambiguous. Thus, S4 was ineffective in reducing primary tumor growth in vivo, although causing decreased spontaneous lung metastases formation in an orthotopic MDA-MB-231 breast cancer model [77]. The S4 involving tests were carried out against MDA-MB-231 triple-negative breast adenocarcinoma, intact (CA IX high) as well as doxycycline-induced CA IX knockdown (CA IX low) HT-29 cells. The authors reported that S4-mediated CA IX inhibition increased DOX efficacy during hypoxia and normoxia exposure in MDA-MB-231 cells, compared to single drug exposure. These results were in line with the lack of S4 efficacy in the HT-29–CAIX low cells. However, contrary to what was expected, DOX sensitivity decreased when HT-29–CA IX low cells were exposed to S4 during hypoxic conditions. Higher serum concentrations also abrogated the effect of S4 on DOX efficacy, which may occur due to the high binding affinity of S4 to bovine serum albumin. MDA-MB-231 tumor-bearing mice were consequently treated with the DOX+S4 combination to evaluate its in vivo efficacy. Disappointingly, S4 co-administration abrogated the effect of DOX in this animal model, and the reasons remained unclear [74].

#### 2.5.5. S4 and Cisplatin

**Figure 13:**
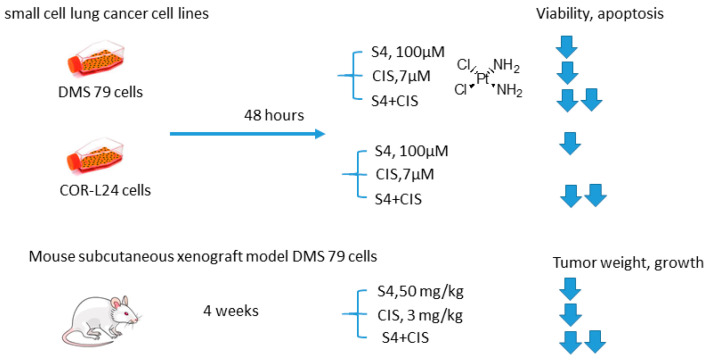
Graphical outline of the study on S4+CIS combination reported by Bryant et al. [74,78].

Soon thereafter, a study on S4+CIS combination against small cell lung cancer emerged (Figure 13) [78]. Bryant and coworkers reported that S4+CIS reduced cell viability in two human small cell lung cancer DMS-79 COR-L24 cell lines. Moreover, in DMS-79 xenograft tumors in nude mice, the S4+CIS combination significantly delayed the tumor growth. Of note is a profound decrease in the necrotic area (almost 50% of tumor area was comprised of necrosis) in the combination therapy group. Dosing with CIS after completion of the 3-week schedule of S4 resulted in a response equal to that of chemo-naive tumors. Therefore, no resistance to therapy was acquired during this treatment. In turn, COR-L24 xenograft tumors showed exquisite sensitivity to S4, keeping tumor sizes at ca. 250 mm^3^ for the 4-week schedule of S4. COR-L24 xenograft tumors were also more CIS responsive than DMS 79, but the treatment was poorly tolerated. In this model, the combination of therapies produced a slightly better treatment response than single agents with COR-L24 tumor regression in three out of four mice showing a strong response. The histological analysis highlighted a significant reduction in CA IX expression and reduced hypoxia regions in COR-L24 tumor xenografts in response to S4 treatment (Figure 13) [78].

#### 2.5.6. *n*-Octyl Disulfamate/SCL-0111 and 3-*O*-acetylbetulin

**Figure 14:**
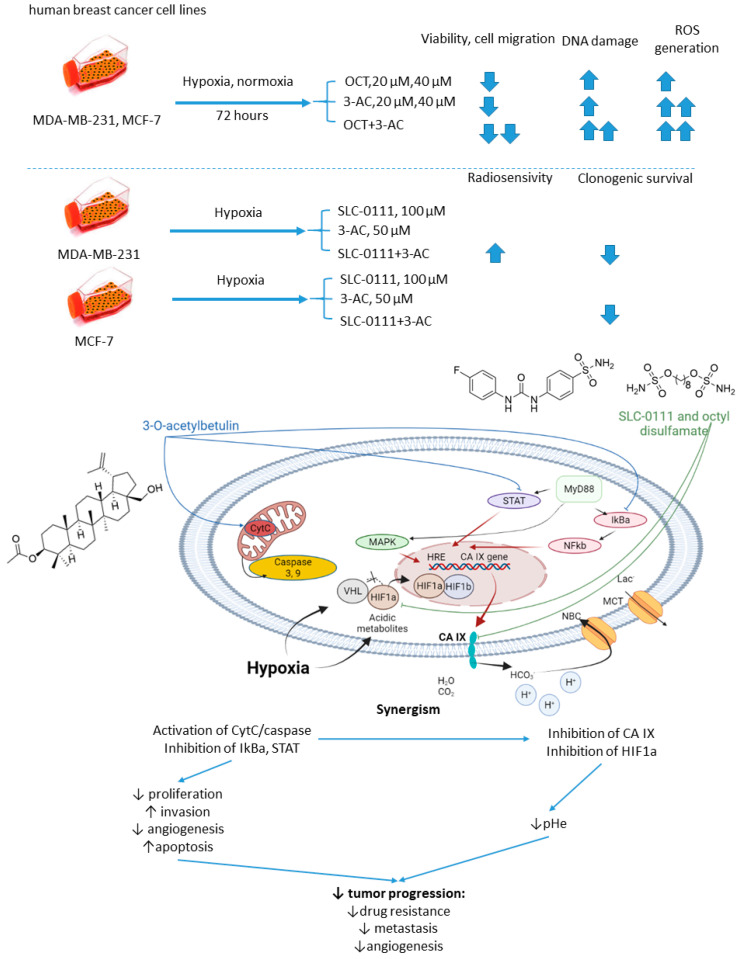
Graphical outline of the study on SLC-0111/OCT+3-AC combination reported by Petrenko et al., and summary on the hypothesized molecular interactions resulting in synergistic action of the drugs [79].

Petrenko and colleagues, in 2021, presented the results of a study on a combination of CA IX inhibitor *n*-octyl disulfamate (OCT) with a pentacyclic triterpene 3-*O*-acetylbetulin (3-AC), a betulinic acid prodrug that shows selective cytostatic activity on human cancer cell lines in vitro and in vivo (Figure 14) [79]. When co-administered, OCT and 3-AC produced remarkable antiproliferative activity and inhibited cell migration in MDA-MB-231 and MCF-7 breast cancer cell lines. Both effects were significantly higher for the combined treatment than the single-drug regimens. Subsequently, SLC-0111 was also tested with 3-AC against breast cancer cells. This drug combination produced a considerable drop in clonogenic survival rates of MDA-MB-231 and MCF-7 cell cultures. Additionally, in MDA-MB-231, but not in MCF-7 cell cultures, increased cells sensitivity towards radiotherapy was observed under exposure to SLC-0111+3-AC treatment (Figure 14) [79].

### 2.6. Isoform Selective Carbonic Anhydrase IX Inhibitors in Combination with Targeted Anticancer Drugs

#### 2.6.1. SCL-0111 and APE1/Ref-1 Inhibitors

Apurinic/apyrimidinic Endonuclease-1/Redox Effector Factor 1 (APE1/Ref-1) is a multifunctional protein possessing a DNA repair function in base excision repair and the ability to reduce oxidized transcription factors, enabling them to bind to their DNA target sequences [80]. APE1/Ref-1 regulates several transcription factors involved in survival mechanisms, tumor growth, redox, and hypoxia signaling [81]. Reports emerged showing that blocking APE1/Ref-1 signaling with selective inhibitor APX3330 leads to decreased activity of STAT3, a drop in HIF1α signaling, and downregulation of CA IX [82].

**Figure 15:**
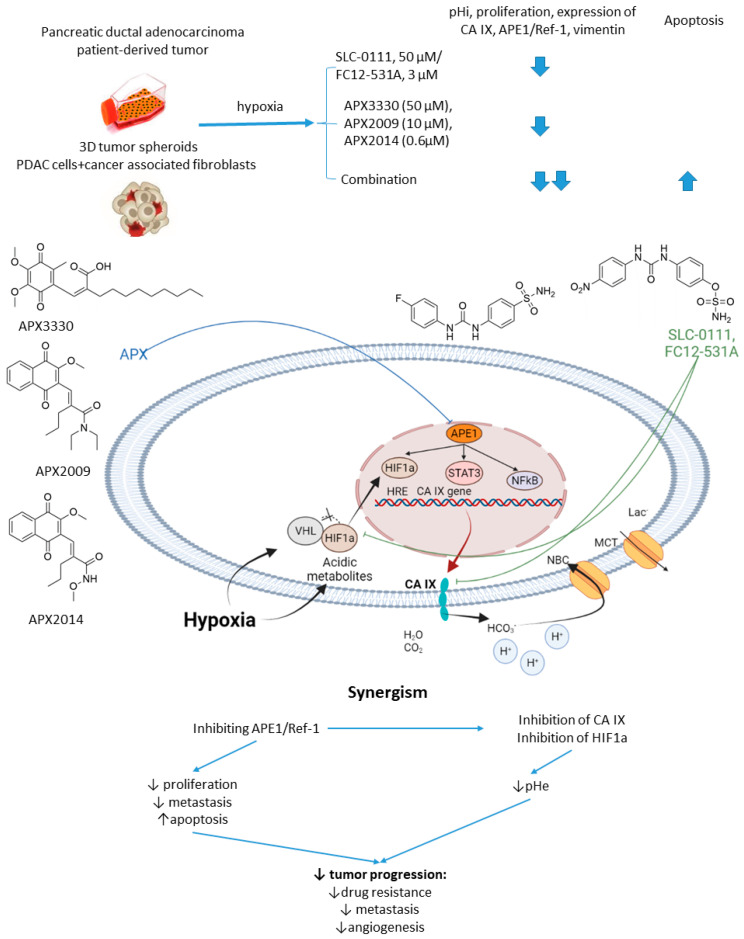
Graphical outline of the study on SLC-0111/FC12-531A+APX3330/APX2009/APX2014 combination reported by Logsdon et al., and summary on the hypothesized molecular interactions resulting in synergistic action of the drugs [84].

In 2016, Logsdon and coworkers investigated the links between APE1/Ref-1 and HIF1α-mediated hypoxia adaptation in pancreatic cancers (Figure 15) [83]. Their in-depth biochemical study culminated in testing APE1/Ref-1 inhibitor APX3330 combined with SLC-0111 against pancreatic tumor spheroids [83]. Thus, dual treatment of hypoxic Panc10.05 pancreatic adenocarcinoma cells resulted in a greater intracellular pH drop and a lower cell survival rate than the monotherapies. Moreover, a significant effect of the SLC-0111+APX3330 combination was corroborated in a 3D co-culture model of pancreatic cancer, including low passage patient-derived tumor cells and cancer-associated fibroblasts. Actually, the tumor-suppressive action of APX3330 was dramatically potentiated in the spheroids, where the cancer cells were selectively killed even when in the protective environment of the fibroblasts [83]. This outstanding work was further continued with a more potent SLC-0111 analog, namely sulfamate-based CAI FC12-531A [84]. Next-generation analogs of APX3330, namely APX2009 and APX2014, were included in the study at that stage. Interestingly, when co-administered with APE1/Ref-1inhibitors, FC12-531A exerted an even higher effect on tumor spheroids than SLC-0111 combinations, resulting in enhanced inhibition of cancer cell growth. Meanwhile, decreased expression of both CA IX and APE1/Ref-1 was observed, orchestrated by a higher caspase-3 positivity in malignant cells (Figure 15) [84].

#### 2.6.2. SCL-0111 and Sunitinib

**Figure 16:**
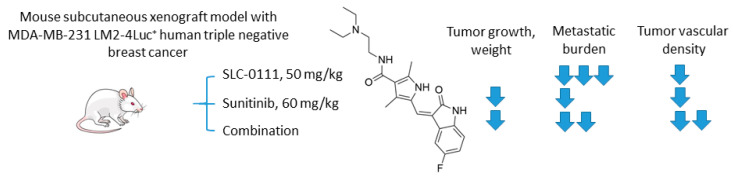
Graphical outline of the study on SLC-0111+sunitinib combination reported by Hedlund et al. [85].

Hedlund and coworkers recently reported the study on the combination of anti-angiogenic tyrosine kinase inhibitor sunitinib and SLC-0111 (Figure 16) [85]. To test the drug combination, the authors employed a highly metastatic MDA-MB-231 LM2-4Luc^+^ in vivo orthotopic model of CAIX-positive human triple-negative breast cancer (TNBC). Interestingly, while sunitinib monotherapy significantly inhibited primary tumor growth, it exacerbated tumor hypoxia and increased metastasis. Of importance, CA IX expression was increased both in the primary tumor and metastases in response to sunitinib exposure. On the other hand, single-agent SLC-0111 treatment resulted in a modest inhibition of tumor growth, albeit dramatically reduced spontaneous metastases. Encouragingly, these two agents, when combined, caused a profound reduction of both tumor volume and metastatic burden in the animal model. The authors subsequently demonstrated that a major result of CA IX blockade was a reduction of the number of blood vessels in the primary tumor, accompanied by a decreased permeability of the remaining vasculature. This is of great significance since sunitinib alone markedly increased permeability of the existing blood vessels, thereby contributing to higher metastasis rates. Moreover, CA IX was expressed by breast tumor cells residing in liver and lung metastases, suggesting additional mechanisms of the observed effect from the addition of SLC-0111. Finally, the study revealed that the genetic depletion of hypoxia-induced CA IX expression by MDA-MB-231 LM2-4Luc+ cells in vitro leads to reduced levels of VEGFA, which may contribute to the observed effects of SLC-0111 on the normalization of tumor vasculature. Summarized, the authors demonstrated a profound inhibition of TNBC tumor growth in vivo, as well as a dramatic reduction in distant metastasis in response to sunitinib+SLC-0111, which was not achievable in the framework of either monotherapeutic regimen (Figure 16) [85].

#### 2.6.3. SCL-0111 and Immune Checkpoint Blockade Inhibitors

**Figure 17:**
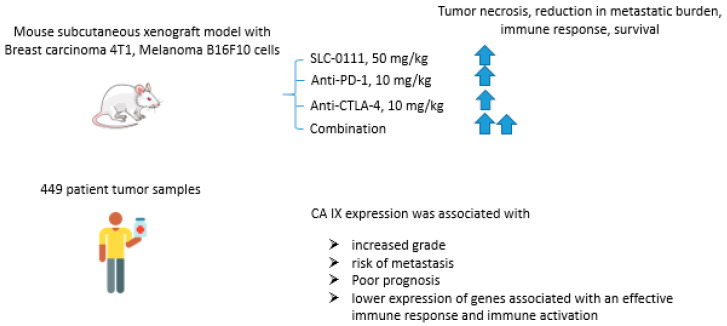
Graphical outline of the study on the combination of SLC-0111+anti-PD-1/anti-CTLA-4 immune checkpoint inhibitors reported by Chafe et al. [88].

Immune checkpoint blockade therapy that prevents cancer cells from escaping a response from tumor-reactive T-cells has recently emerged as a highly promising approach in cancer management [86]. In fact, CTLA-4 (cytotoxic T-lymphocyte-associated protein 4), PD-1 (programmed cell death protein 1), and its ligand PD-L1 inhibitors proved to interfere with T-cell inhibitory pathways, thereby overcoming tumor immune subversion [87]. Considering that tumor acidosis is known to be a significant burden for immune functions, Chafe and coworkers reported an expanded study on the potential of CA IX inhibition to enhance the immune checkpoint blockade effect in cancer (Figure 17) [88]. Thus, in vitro tests with co-culture presented by murine skin melanoma B16F10 cells and activated T-cells, the research group showed that the use of SLC-0111 (100 μM) increased T-cell antitumor response. The results obtained were confirmed in animal models with subcutaneous mammary adenocarcinoma 4T1 and skin melanoma B16F10 cells. In both models, the combination of SLC-0111 and anti-PD1/anti-CTLA4 agents reduced the number of T regulatory cells (Tregs) and T-helper 17 cells (Th17), increased the number of Th1, CD8+ cells in tumor tissue and granzymes production by B-cells. Moreover, the drug combination contributed to tumor necrosis, reduction in metastatic burden and increased the survival of animals in comparison with monotherapy. This study was followed by the analysis of patient tumor samples (*n* = 449), revealing the association of CA IX expression with the increased grade, risk of metastasis, and generally poor prognosis. The authors also found that increased CA IX expression correlates with lower expression of genes associated with an effective immune response and immune activation, such as CD3E, CD8A, and CD4. The obtained results, therefore, call attention to the important effect of CA IX activity on antitumor immune response and the potential to enhance immune checkpoint therapy outcomes via pharmacologically blocking this latter enzyme (Figure 17) [88].

#### 2.6.4. SCL-0111 and Suberoylanilide Hydroxamic Acid

**Figure 18:**
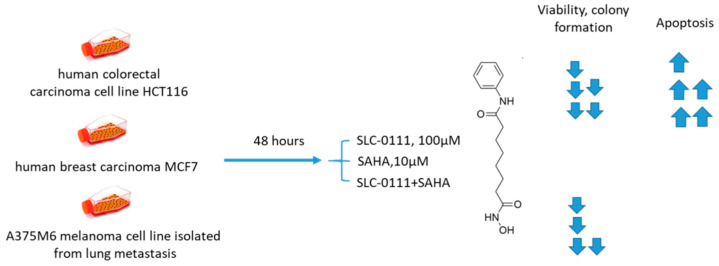
Graphical outline of the study on SLC-0111+SAHA combination reported by Ruzzolini et al. [90].

Ruzzolini and coworkers recently investigated the joint in vitro antiproliferative action of suberoylanilide hydroxamic acid (SAHA), the first FDA-approved HDAC inhibitor, and SLC-0111 (Figure 18) [89,90]. The SLC-0111+SAHA combination exhibited enhanced antiproliferative effects against HCT116 colorectal carcinoma, MCF7 breast carcinoma, and A375M6 melanoma cell lines. The degree of potentiation was especially high in HCT116 and MCF7 cells, whereas A375M6 cells were least sensitive to the treatment. A remarkable increase of cells in the G2/M phase in HCT116 and MCF7 cell lines, but not in A375M6, was observed. In turn, colony formation was almost completely abrogated by the SLC-0111+SAHA combination in the tested cell lines and the most remarkable synergistic effect was observed in HCT116 cells. Similarly, when acetylation of histone H4 was assessed, as well as that of non-histone HDACs target p53, it turned out that SLC-0111 considerably increased the acetylation levels in both targets. In HCT116 cells, this effect was shown to be associated with enhanced poly(ADP-ribose) polymerase (PARP) cleavage and apoptosis, which was not true for MCF7 and A375M6. Therefore, the combination of SAHA and SLC-0111 proved efficient against three cancer cell lines, and HCT116 showed a profound and mechanism-based response (Figure 18) [90].

#### 2.6.5. S4/FC9–399A and Proton Pump Inhibitors

**Figure 19:**
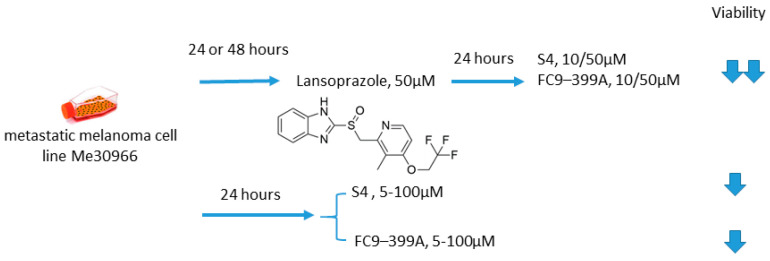
Graphical outline of the study on S4/FC9–399A +lasoprazole combination reported by Federici et al. [13].

Growing evidence exists that proton pump inhibitors (PPIs) can be beneficial in cancer treatment, allowing for suppression of tumor metabolic adaptation to hypoxia and overcoming pH-driven drug resistance [91,92,93]. Of note, contrary to the vast majority of anticancer drugs, PPIs protonation in an acidic environment leads to activation instead of neutralization [94]. Federici and colleagues, in 2016, undertook an intriguing investigation on the synergistic antitumor action of a PPI lansoprazole and CA IX inhibitors S4 and FC9–399A, as simultaneous blocking of the most important proton exchangers appeared promising for the management of solid tumors (Figure 19) [17]. Within the study, the metastatic melanoma Me30966 cell line was cultured in an unbuffered medium, allowing for spontaneous culture medium acidification by tumor cells. Interestingly, in such conditions, preincubation with lansoprazole leads to a significant increase in the anticancer activity of both S4 and FC9–399A. In fact, a dramatic drop in cell survival was observed in the presence of lansoprazole, whereas each of the single-drug treatments exerted a neglectable effect on Me30966 viability. While spontaneous acidification was suitable for a full activation of lansoprazole, protein pump inhibition could prevent S4 and FC9–399A acidification, resulting in the promising in vitro data for the suggested drug combinations [95]. Therefore, it was supposedly not the CA IX inhibitor in this case that sensitized the cells, but instead, lansoprazole that induced the favorable conditions for the S4 and FC9–399A pharmacological action (Figure 19) [95].

## 3. Discussion and Perspectives

The literature analyses indicate significant progress in deciphering the potential of CA IX inhibitors as adjuvant cancer treatments, with more than two dozen research articles published in recent years. These reports comprised preliminary 2D cell culture tests as well as in-depth ex vivo and in vivo studies simulating the complex tumor microenvironment, protective mechanisms, and tumor–host immune interactions.

**Figure 20:**
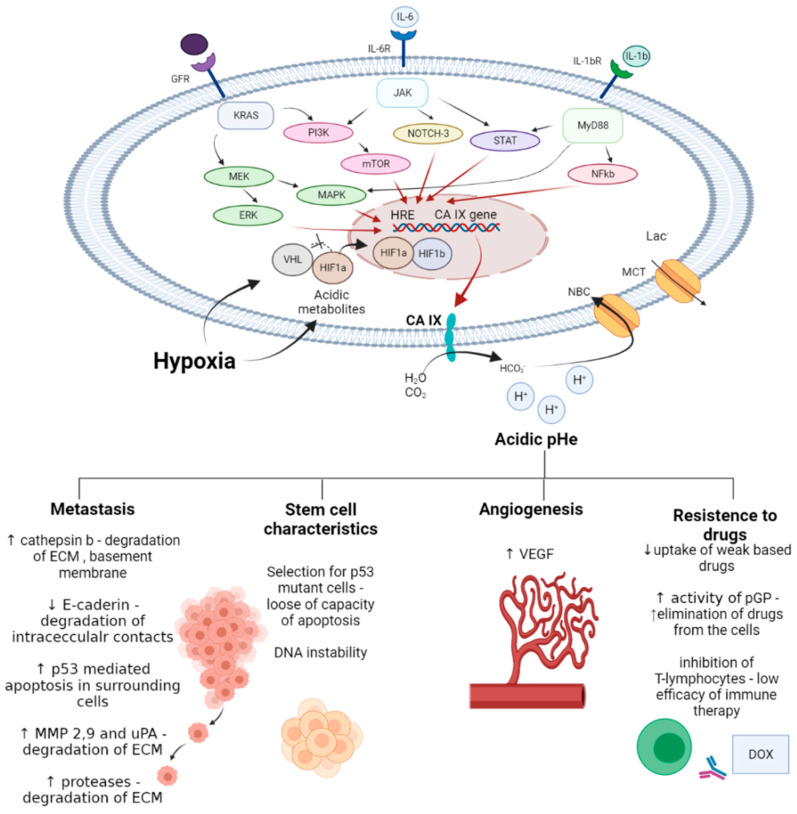
General considerations on the mechanisms of anticancer drug potentiation by CA IX inhibition in cancer cells.

Early works demonstrated that CA IX inhibitors are capable of enhancing the bioavailability of weakly basic drugs, which suffer from regional failure in acidic tumor compartments. Subsequently, a series of thorough and well-designed investigations revealed the appreciable potential of pharmacological CA IX blockades to intensify cancer therapies and improve treatment outcomes in many malignancies. In fact, such reports described enhanced cytotoxic action of the drugs due to pH_i_ alterations and apoptosis induction in cancer cells. Moreover, co-administration of CA IX inhibitors prevented malignant cell invasion, matrix destruction and metastasis, and inhibited angiogenesis in animal models. Finally, the mechanism-based synergy of CA IX inhibitors with chemo and immunotherapeutic agents has been unraveled. It was demonstrated that the effects of drugs affecting GFR (grow factor receptor)/PI3K/Akt/mTOR, GFR/MEK/ERK, IL-6R/JAK (Janus kinase)/NOTH-3, IL-1βR/MyD88 (myeloid differentiation primary response gene (88))/STAT (signal transducer and activator of transcription proteins)/ NF-kB (nuclear factor kappa-light-chain-enhancer of activated B cells), and other crucial signaling pathways that activate HIF1α and HRE can be significantly potentiated by blocking CA IX enzymatic activity (Figure 20).

Being widely used in clinical settings, pan-isoform inhibitors acetazolamide and methazolamide demonstrated significant efficacy in combination therapy of solid tumors. As non-type-specific CA blockers, these drugs may also benefit from inhibiting other CA isoforms, which roles in cancer may be under-evaluated at the current stage [30]. On the other hand, a high affinity towards ubiquitous cytosolic CA I and II isoforms may play a detrimental role leading to suboptimal drug distribution profiles and undesired effects [30].

Importantly, with its successfully finished safety studies within the clinical trials, SLC-0111 was effectively employed in various combinations with anticancer drugs. The potential of this adjuvant agent will be crystallized upon Phase II completion in the coming years [96]. Meanwhile, other CAIX inhibitory drug candidates possessing different isoform selectivity and PK profiles are of great interest to further establish the potential of CA IX blockade in tumors and boost the discovery of highly efficient adjuvant agents targeting the tumor microenvironment.

Despite this success in the in vitro and in vivo experiments, the capabilities of different tumors to adapt to the combination therapy have not been properly established at the current stage. In fact, only a few reports contained posttreatment survival data for the investigated drug combinations. Particularly, Faes et al. [45] communicated the mice tumor xenografts did not progress after 3 months of AZ+rapamycin treatment; however, the decrease in the tumor volumes was not observed. This indicates that there is still a potential for resistance against the suggested drug combination. On the other hand, the SLC-0111 produced 100% survival rates when combined with temozolomide or gemcitabine (130 days posttreatment) [68,97].

**Figure 21:**
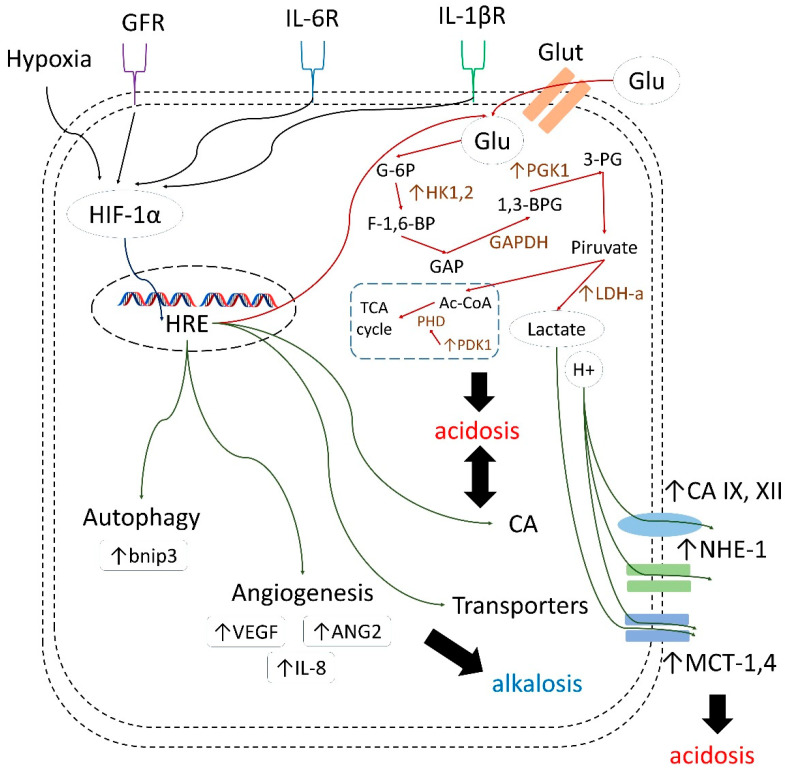
HIF-1α/HRE-mediated adaptation of cancer cells to tumor acidosis [98,99,100,101,102].

Moreover, along with further investigations of drug combinations discussed in the present review, significant benefit can be expected from co-administrating CA IX inhibitors with different agents that affect various HIF-induced cellular processes, including autophagy, angiogenesis, and glycolysis. In fact, considering the role of these latter processes in tumor cell growth and survival, there is great room for synergistic action of such combinations (Figure 21) [98,99,100,101,102].

Furthermore, the selection of drug combinations involving CA IX inhibitors can be guided by gene-knockout experiments unveiling synthetic lethal gene couples in cancer cells [103]. Very recently, Chafe et al. revealed a group of genes networks that are significantly affected by the loss of CA IX, associated with:

- the cytoskeleton;
- cell cycle and mitosis, ribosome biogenesis, RNA processing, DNA damage repair, and nucleic acid metabolism;
- mitochondrial organization;
- redox homeostasis: thioredoxin, glutathione S-transferases, glutathione reductase, and the molybdenum cofactor and iron-sulfur cluster biogenesis: the cysteine desulfurase (NFS1) and iron-sulfur cluster scaffold protein;
- iron-sulfur cluster adapter protein, adenosine 5′-triphosphate–binding cassette subfamily B member 7, and glutaredoxin 5 [103].

**Figure 22:**
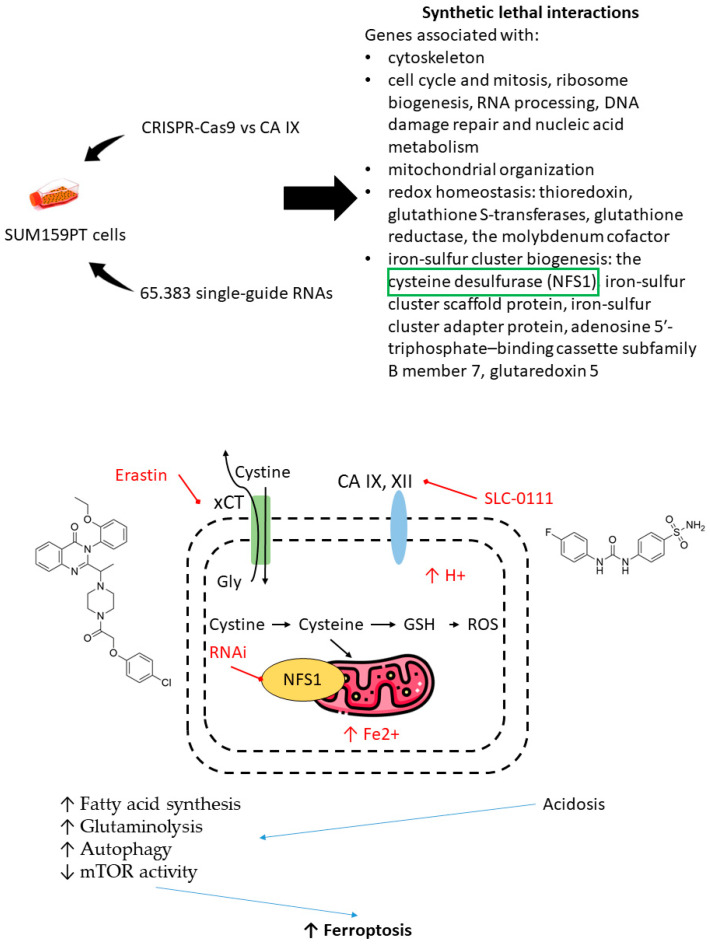
Graphical outline of the study on synthetic lethal gene interactions reported by Chafe et al. [103].

One of the most significant synthetic lethal genes turned out to be NFS1-encoding cysteine desulfurase. This mitochondrial enzyme catalyzes the generation of cofactors containing iron for proteins, involved in a number of cellular functions. In addition, cysteine desulfurase plays an important role in protecting cells from ferroptosis. Chafe and coworkers demonstrated a synergic increase of cell death due to ferroptosis after co-administration of SLC-0111 and siRNA suppressor of NFS1/non-lethal doses of erastin or sulfasalazine (the cystine glutamate antiporter xCT). SLC-0111 contributed to intracellular acidosis, which increases susceptibility to ferroptosis, fatty acid synthesis, glutaminolysis, and autophagy and decreases mTOR activity (Figure 22) [103].

These results, again, highlighted that CA IX inhibitors can significantly affect the pathogenic mechanisms with common signaling pathways and gained attention for the new targets for combination therapy, which were discovered via genome-wide synthetic lethal screening [104].

Summarized, CA IX inhibition proved to significantly impact overcoming tumor resistance against conventional cytotoxic agents by reducing poorly accessible hypoxic regions and extracellular acidosis, orchestrated by intensifying the treatment-induced pHi acidification. In the meantime, the remarkable potential of combining CA IX inhibitors with targeted anticancer drugs, especially those affecting HIF/HRE-related signaling pathways, has been highlighted by several in-depth studies employing complex and advanced tumor models. A few crucial directions for further efforts can be highlighted, such as; (1) enriching the currently available CAIs arsenal with highly CA IX-specific molecules possessing favorable stability and tissue distribution, (2) identifying more drugs that can be potentiated by co-administration of CA IX inhibitors according to the pathogenic mechanism, resistance mechanisms, and lethal gene interactions, (3) investigating the drug combinations in 3D-models, and properly simulating tumor microenvironments, and (4) developing well-justified protocols for in vivo studies, including posttreatment survival analysis and knockout animals to observe the resistance mechanisms. Finally, it should be noticed that tumor microenvironment traits remain decisive in the pathogenesis of many cancer phenotypes and in this context, tumor-specific and readily druggable enzyme CA IX represents an attractive drug target for innovative therapeutic approaches.

## Figures and Tables

**Table 1:** CA inhibitory agents evaluated for combination cancer treatment, their Ki values against therapeutically relevant enzyme isoforms and general information on the current status.

Drug/Drug Candidate	Chemical Structure	K_i_ (nM)		Current Status	Ref
Acetazolamide (AZ)	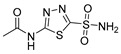	CA I	250	systemic CAI clinically used to treat glaucoma, edema, certain types of epilepsy	[13,14]
		CA II	12		
		CA IX	25		
		CA XII	5.7		
Methazolamide	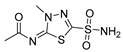	CA I	50	systemic CAI clinically used to treat glaucoma	[13,14]
		CA II	14		
		CA IX	27		
		CA XII	3.4		
SLC-0111	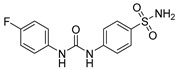	CA I	5080	in clinical trials as adjuvant agent in advanced solid tumorsPhase Ib/II	[15]
		CA II	960		
		CA IX	45		
		CA XII	4.5		
*n*-Octyl sulfamate	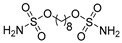	CA I	378	rarely used tool compound for in vitro experiments	[16]
		CA II	15		
		CA IX	4		
		CA XII	-		
S4	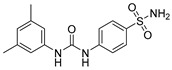	CA I	5600	in preclinical studies	[17]
		CA II	546		
		CA IX	7		
		CA XII	2		
FC-531A	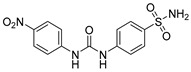	CA I	1230	in preclinical studies	[17]
		CA II	450		
		CA IX	6		
		CA XII	4		

## Abbreviations

1,3-BPG	1,3-Bisphosphoglycerate
3-PG	3-phosphoglyceric acid
Ac-CoA	acetyl coenzyme A
Akt (PKB)	protein kinase B
ANG2	angiopoietin 2
APE1/Ref-1	apurinic/apyrimidinic endonuclease-1/redox effector factor 1
AZ	acetazolamide
Bnip3	Bcl-2/adenovirus EIB 19-kD interacting protein 3
BTIC	brain tumor initiating cell
CA	carboanhydrase
CA IX	carbonic anhydrase IX
CAIs	CA inhibitors
CHOP	cyclophosphamide, doxorubicin hydrochloride hydroxydaunorubicin, vincristine sulfate Oncovin, and prednisone
CIS	cisplatin
CTLA-4	cytotoxic T-lymphocyte-associated protein 4
DOX	doxorubicin
EAC	Ehrlich ascites carcinoma
EGFR	epidermal growth factor receptor
ERK	extracellular signal-regulated kinase
EV1	empty vector
F-1,6-BP	fructose-1,6-bisphosphate
G-6P	glucose -6 phosphate
GAP	glyceraldehyde-3-phosphate
GAPDH	glyceraldehyde 3-phosphate dehydrogenase
GFR	growth factor receptor
GLU	glucose
GLUT	glucose transporter
GSH	glutathione
HDAC	histone deacetylases
HIF-1a	hypoxia-inducible factor 1a
HK	hexokinase
HRE	hypoxia response element
HUVEC	human umbilical vein endothelial cells
IL	interleukin
IM	imatinib
JAK	Janus kinase
KEAP1	Kelch Like ECH Associated Protein 1
KRAS	Kirsten rat sarcoma
LDH-a	lactic dehydrogenase-a
MAPK	mitogen activated protein kinase
MCT	monocarboxylate transporters
MEK	MAPK/ERK kinase
MMP	matrix metalloproteinases
mTOR	mechanistic target of rapamycin
mTORC	mTOR complexe
MyD88	myeloid differentiation primary response gene (88)
NF-kB	nuclear factor kappa-light-chain-enhancer of activated B cells
NFS1	iron-sulfur cluster biogenesis: the cysteine desulfurase
NHE	Na+,H+-exchanger
OCT	n-octyl disulfamate
PARP	poly(ADP-ribose) polymerase
PD-1	programmed cell death protein 1
PDGFR	platelet-derived growth factor receptor
PDK1	pyruvate dehydrogenase kinase 1
PGK1	phosphoglycerate kinase 1
PHD	prolyl hydroxylases
pHe	extracellular pH
pHi	intracellular pH
PI3K	phosphatidylinositol-3 kinase
PPIs	proton pump inhibitors
ROS	reactive oxygen species
SAHA	suberoylanilide hydroxamic acid
SFN	sulforaphane
STAT	signal transducer and activator of transcription proteins
TCA	tricarboxylic acid
Th17	T-helper 17 cells
TIMP-1	tissue inhibitor of metalloprotease-1
TNBC	triple-negative breast cancer
Tregs	T regulatory cells
VEGF	vascular endothelial growth factor
VHL	Von Hippel-Lindau
